# Enhancement of Asynchronous Release from Fast-Spiking Interneuron in Human and Rat Epileptic Neocortex

**DOI:** 10.1371/journal.pbio.1001324

**Published:** 2012-05-08

**Authors:** Man Jiang, Jie Zhu, Yaping Liu, Mingpo Yang, Cuiping Tian, Shan Jiang, Yonghong Wang, Hui Guo, Kaiyan Wang, Yousheng Shu

**Affiliations:** 1 Institute of Neuroscience, State Key Laboratory of Neuroscience, Shanghai Institutes for Biological Sciences, Chinese Academy of Sciences, Shanghai, China; 2 Department of Neurosurgery, Shanghai Quyang Hospital, Tongji University, Shanghai, China; 3 Department of Neurology, Huashan Hospital, Fudan University, Shanghai, China; Institut du Cerveau et de la Moelle épinière Hôpital Pitié-Salpêtrière, France

## Abstract

Down-regulation of GABAergic inhibition may result in the generation of epileptiform activities. Besides spike-triggered synchronous GABA release, changes in asynchronous release (AR) following high-frequency discharges may further regulate epileptiform activities. In brain slices obtained from surgically removed human neocortical tissues of patients with intractable epilepsy and brain tumor, we found that AR occurred at GABAergic output synapses of fast-spiking (FS) neurons and its strength depended on the type of connections, with FS autapses showing the strongest AR. In addition, we found that AR depended on residual Ca^2+^ at presynaptic terminals but was independent of postsynaptic firing. Furthermore, AR at FS autapses was markedly elevated in human epileptic tissue as compared to non-epileptic tissue. In a rat model of epilepsy, we found similar elevation of AR at both FS autapses and synapses onto excitatory neurons. Further experiments and analysis showed that AR elevation in epileptic tissue may result from an increase in action potential amplitude in the FS neurons and elevation of residual Ca^2+^ concentration. Together, these results revealed that GABAergic AR occurred at both human and rat neocortex, and its elevation in epileptic tissue may contribute to the regulation of epileptiform activities.

## Abstract 2

Asynchronous GABA release occurs at output synapses of fast-spiking interneurons in human and rat neocortex and is elevated in epileptic tissues from both species.

## Author Summary

The balance between excitation and inhibition in the cerebral cortex is important for multiple brain functions. Down-regulation of GABA-induced inhibition disrupts this balance and may lead to epileptic seizures. Asynchronous release of GABA is known to occur at certain GABAergic synapses and represents release of inhibitory neurotransmitter that is not precisely timed to presynaptic action potentials. Whether asynchronous release is subject to change after the induction of epilepsy remains unclear. In this study, using simultaneous recordings from inhibitory fast-spiking neurons and excitatory pyramidal cells, we found that asynchronous release occurred at the output synapses of fast-spiking neurons in both human and rat neocortex. The occurrence of asynchronous release depended on the level of residual calcium at the presynaptic terminals but not on postsynaptic spiking. Further experiments using cortical tissue derived from human patients with intractable epilepsy and from a rat model of the disorder revealed an elevation of asynchronous release in epileptic cortex, possibly resulting from an increase in action potential amplitude of fast-spiking neurons and changes in calcium dynamics in their axon terminals. Taken together, these results demonstrate that asynchronous release is a fundamental property shared by neocortical fast-spiking neurons regardless of species, and the enhancement of asynchronous release in epileptic tissue suggests a role for it in regulating epileptic activities.

## Introduction

During active states in the cerebral cortex, cortical neurons receive both excitatory and inhibitory synaptic inputs. Proper balance of these inputs [1],[2] is important for neuronal responsiveness to incoming inputs [3],[4] and for sensory processing [5],[6]. Disruption of this balance may cause malfunctioning of the network, leading to various brain disorders such as epileptic seizures [7],[8]. The main inhibitory neurotransmitter in the cortex is GABA, which is normally released from axonal terminals of inhibitory interneurons and mainly activates GABA_A_ and GABA_B_ receptors, leading to cortical inhibition [9]. The balance between excitation and inhibition largely depends on proper regulation of the activities of these interneurons and the excitatory pyramidal cells (PCs) [10]–[13].

Molecular and functional changes in GABA receptors [14],[15] or selective loss [16]–[20] or dormancy [21]–[23] of inhibitory interneurons may result in hyperexcitability of neuronal networks and contribute to epileptogenesis. However, there are also several lines of evidence showing no substantial change in the basal GABAergic transmission in epileptic tissues [24]–[27]. It is possible that other changes in the properties of inhibitory synapses associated with high-frequency discharges may be involved in generating and regulating the network activities, including the epileptiform activity.

Under most circumstances, action potential (AP) is initiated at the axon initial segment and propagates to the presynaptic terminals, triggering neurotransmitter release within milliseconds [28]. This tightly coupled or synchronized transmitter release with presynaptic AP generation ensures precise signaling in the complex neural network. However, prolonged asynchronous release (AR) for hundreds of milliseconds following presynaptic AP burst has been observed at some excitatory and inhibitory synapses, particularly after high-frequency firing of presynaptic neurons [29]–[32]. At GABAergic synapses, AR may provide long-lasting inhibition and reduce the discharge probability and precision in postsynaptic neurons, leading to desynchronization of network activities. A recent study demonstrated that, after a burst of APs, fast-spiking (FS) interneurons in the rat neocortex show AR at their output synapses, including FS autapses and FS-PC synapses [33]. AR at FS autapses results in self-inhibition and consequently excitation of its target cells, while that at FS-PC synapses causes inhibition of target PCs. Therefore, regulation of the AR-induced self-inhibition in FS neurons and inhibition in PCs may contribute to the proper excitation-inhibition balance in the cerebral cortex. In this study, we examine whether AR occurs in human epileptic neocortical tissue and whether AR is subjected to change after the induction of epileptic seizures.

We obtained human cortical tissues from small brain blocks that were surgically removed to cure intractable epileptic patients and brain tumor patients. Since the surgery is considered a therapy of the last resort for patients that had frequently suffered severe epileptic seizures, the cortical tissue should have experienced chronic epileptiform activities. We found that although AP burst-evoked AR occurred in all GABAergic synapses of FS interneurons (including FS autapses, FS-FS and FS-PC synapses) in these human epileptic tissues, FS autapses exhibited the strongest AR among these synapses. Further experiments in rats revealed similar differences in AR at different synapses. Importantly, as compared with control tissues, AR is significantly stronger in epileptic tissues, indicating that AR at GABAergic synapses might be subjected to modulation by epileptic seizures and involved in regulating epileptiform activities.

## Results

### AR at FS Autapses and FS-FS Synapses in Human Cortical Slices

Human neocortical tissues from 52 patients (aged 5–42 y) with frontal or temporal lobe epilepsy were sliced and examined by electrophysiology within 2–10 h after surgical removal. Whole-cell recording was performed on single FS neurons or synaptically connected FS-FS and FS-PC pairs in layer 5.

**Figure 1:**
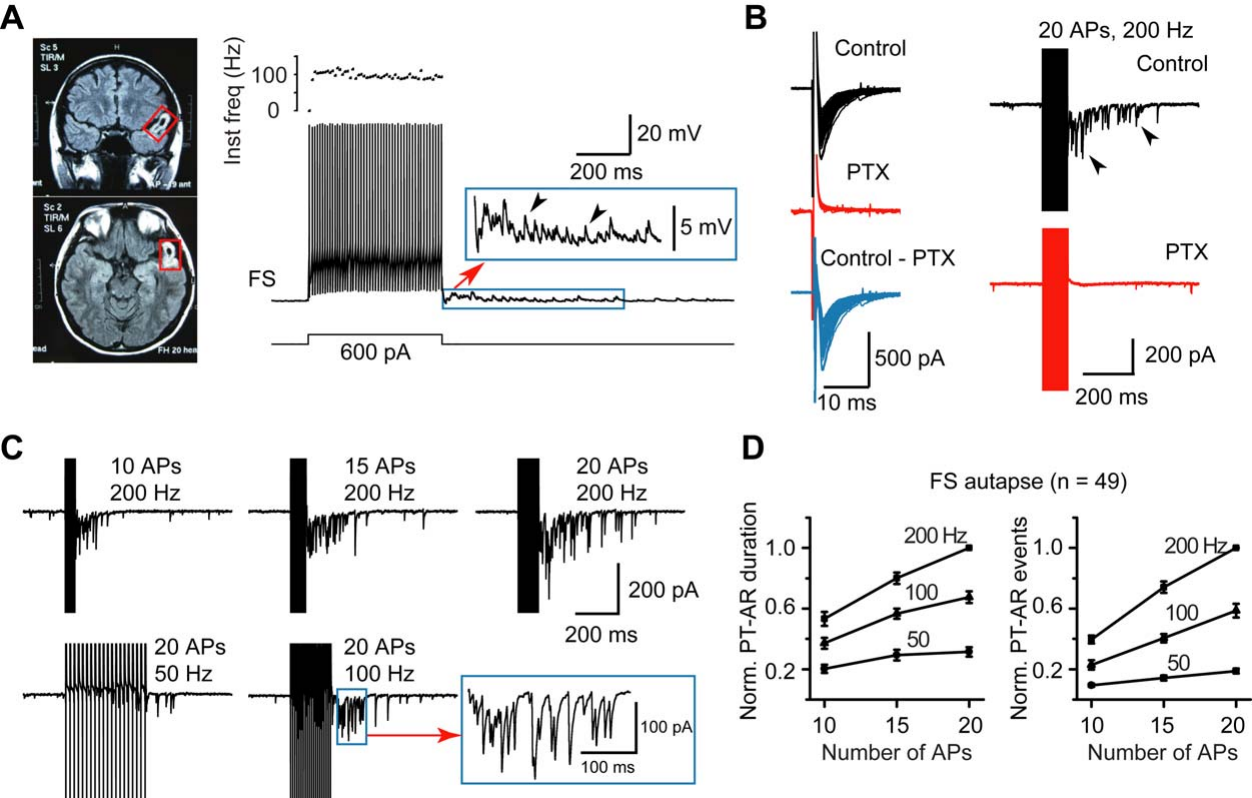
AR in FS autapses in human epileptic neocortical tissue. (A) Left, magnetic resonance imaging (MRI) of a patient's brain with glioma (red boxes) in the temporal lobe. This patient also showed severe epileptic seizures. Top, coronal plane; bottom, horizontal plane. Right, typical firing pattern of an FS neuron obtained from the periglioma tissue. Step current injection evoked a train of APs at ∼100 Hz without adaptation. Note the increase in spontaneous events after the AP train (inset). Pipette solution contained 75 mM Cl^−^. (B) Both single AP-evoked synchronous release (left) and AP burst-evoked PT-AR (right) in the FS neuron could be blocked by GABA_A_ receptor blocker picrotoxin (PTX, 50 µM). Single APs were evoked by brief (0.3∼0.5 ms in duration) step commands from −70 to 40 mV. (C) Example current traces showing the dependence of PT-AR duration and total number of events on the number and the frequency of APs. Inset, expansion for clarity. (D) Group data (two-way ANOVA analysis, *p*<0.001 for both AP frequency and number).

We first examined the properties of asynchronous release (AR) of GABA at autapses made by single FS neurons on themselves. In about 22% of FS neurons tested (*n* = 85/392), we consistently observed elevated spontaneous synaptic events immediately after high-frequency firing evoked by DC current injection through the recording pipette in current-clamp mode (Figure 1A). By using a high-Cl^−^ pipette solution (75 mM Cl^−^), inhibitory postsynaptic potentials (IPSPs) were depolarizing events at the resting membrane potential (∼−70 mV). Consistent with previous findings in rodents [34], we found that in voltage-clamp mode (V_hold_ = −70 mV) single AP could trigger an inward current in the same recorded cell that peaked within 2 ms and could be completely blocked by the bath application of picrotoxin (PTX, 50 µM; *n* = 15), a GABA_A_ receptor antagonist (Figure 1B). This indicates the existence of monosynaptic autaptic connections in these human FS interneurons. These unitary inhibitory postsynaptic currents (IPSCs) had a failure rate of 0.3±0.3% and an onset latency of 0.84±0.07 ms; the rise time and decay time constant were 0.59±0.08 and 3.9±0.5 ms, respectively (*n* = 12 FS neurons). The amplitudes of these IPSCs were relatively large (255.8±53.6 pA) because we selectively examined the effects of PTX on FS neurons with obvious autaptic unitary IPSCs, ensuring accurate measurements of the IPSC kinetics after subtraction (control – PTX, Figure 1B). In another set of recordings, we applied PTX for every FS neuron recorded to examine the probability of autaptic connections; we found that 9/14 cells (64.3%) had autaptic synapses, slightly less than that found in rodents [34].

Similarly, in voltage-clamp mode, the spontaneous events following trains of high-frequency stimulation (>50 Hz) in FS neurons were also completely abolished by PTX, indicating that these events were also GABA_A_ receptor-mediated spontaneous IPSCs (sIPSCs) (Figure 1B). In physiological conditions, GABAergic responses normally hyperpolarize postsynaptic neurons, and therefore it is unlikely that FS neuron firing could drive other neurons to fire APs [13],[35],[36]. Thus, these sIPSPs or sIPSCs are unlikely to be caused by recurrent polysynaptic events but rather are attributable to AR at autapses. In response to a train of stimulation (20 APs at 200 Hz), post-train AR (PT-AR) lasted for 187±11 ms and had a total number of 17.7±1.6 spontaneous events (*n* = 74, Figure 1C; see Materials and Methods). We then varied the stimulation frequency and the number of APs and found that the duration and event number of PT-AR progressively increased with increasing stimulus intensity (two-way ANOVA analysis, *p*<0.001 for both AP frequency and number, *n* = 49; Figure 1C–D). Due to the difficulties of identifying individual AR events among action currents (FS autapses) or synchronous IPSCs (FS-FS and FS-PC synaptic connections, shown below) that occurred during the high-frequency train stimulation, we only analyzed the properties of PT-AR in this study. Together, these results revealed the existence of robust AR at FS autapses in human epileptic neocortical tissue, indicating a long-lasting self-inhibition of FS neurons during high-frequency firing.

**Figure 2:**
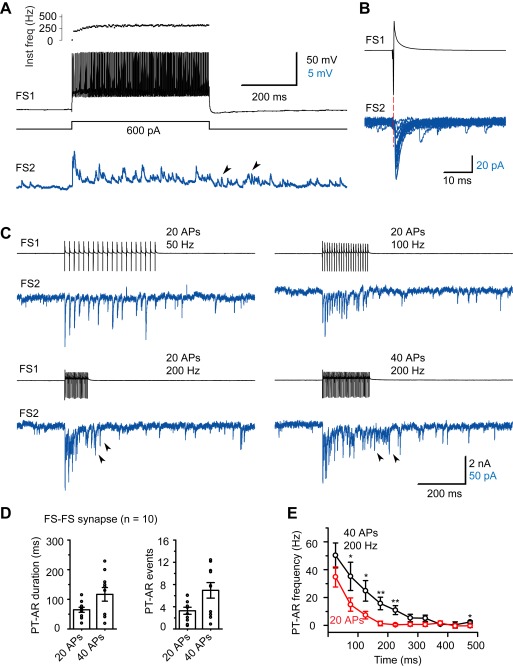
AR in FS-FS synapses in human cortical tissue. (A) Example FS-FS pair. Step current injection induced high-frequency discharges in the presynaptic FS neuron and synaptic events in the postsynaptic FS neuron. Arrowheads indicate the AR events after the stimulus. (B) Same pair as in (A). Overlay of unitary IPSCs evoked by single APs. (C) Example current traces showing the dependence of AR on the number and the frequency of presynaptic APs. (D) Group data of FS-FS pairs. (E) Plot of PT-AR frequency (bin size: 50 ms) as a function of time since the stop of stimulus (20 or 40 APs at 200 Hz). * *p*<0.05; ** *p*<0.01.

In addition to their autaptic connections, FS neurons also form inhibitory synaptic connections onto other FS neurons (Figure 2A–B). We found chemical synapses in 12/39 (30.8%) FS-FS pairs (intersomatic distance<50 µm), including 11 uni-directional connections and one bi-directional connection. Among these 39 pairs, nine pairs (23.1%) showed electrical coupling and three pairs (7.7%) were both chemically and electrically connected. The unitary IPSCs had a failure rate of 1.3±0.5%, an average peak amplitude of 58.1±13.5 pA and an onset latency of 0.95±0.11 ms; the rise time and decay time constant were 0.78±0.03 and 4.5±0.6 ms, respectively (*n* = 11). We found that high-frequency firing in presynaptic FS neurons also evoked long-lasting AR, detected as sIPSCs in the postsynaptic FS neuron (*n* = 10/10 pairs, Figure 2C). Similarly, AR at FS-FS synapses depended on the intensity of presynaptic stimulation. Increasing the frequency or the number of presynaptic APs increased the duration and total number of PT-AR (Figure 2C–D). The average duration and number of events were 64.8±8.6 ms and 3.2±0.6, respectively, in response to 20 APs at 200 Hz in presynaptic FS neurons and increased to 117±23 ms (paired *t* test, *p*<0.01) and 7.0±1.4 (*p*<0.01) when the number of APs were increased to 40 (*n* = 10 FS-FS pairs; Figure 2D). The PT-AR frequency was also significantly increased (Student's *t* test; Figure 2E). Interestingly, in comparison with FS autapses, both the duration and the number of PT-AR were significantly smaller in FS-FS synapses (K-S test, *p*<0.001). Together, these results indicate that although weaker than in FS autapses, asynchronous GABA release also occurs in synaptically connected FS neurons, providing long-lasting inhibition within this population of cortical inhibitory neurons.

### AR in FS-PC Synapses in Human Cortical Slices

**Figure 3:**
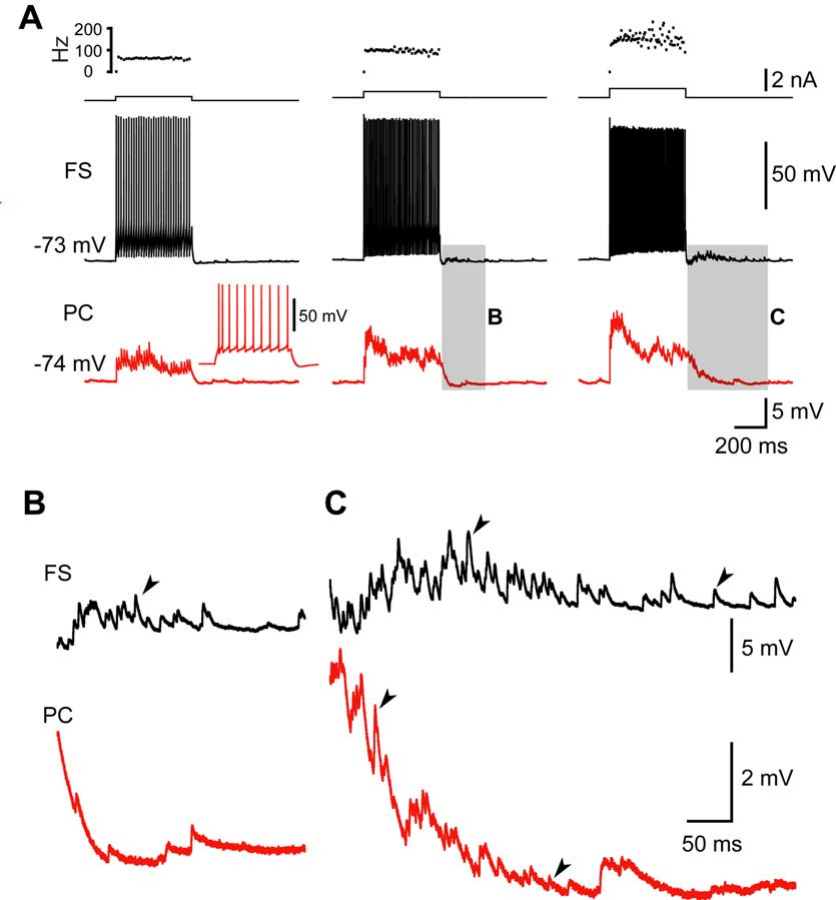
AR in FS-PC synapses in human cortical tissue. (A) Example FS-PC pair recording. In current-clamp mode, trains of APs in presynaptic FS neuron (black) triggered not only autaptic sIPSPs but also synchronous IPSPs and prolonged asynchronous IPSPs in the postsynaptic PC (red). Inset, regular firing pattern of the PC. (B,C) Expansion of the traces in the shadowed boxes shown in (A). Note that FS autapses had longer PT-AR duration and more events than FS-PC synapses. Arrowheads indicate single AR events.

Next, we investigated the occurrence of AR in FS synapses onto excitatory PCs (Figure 3). Again, after bursts of APs in the presynaptic FS neuron, we frequently observed AR during and following the AP burst-triggered synchronous release (*n* = 61/66 pairs). In current-clamp mode, a train of APs evoked by step current injection in FS neuron evoked both autaptic (through FS autapses) and synaptic (through synapses from FS neuron to PC) AR in a stimulus intensity-dependent manner (Figure 3A). Close examination of these PT-AR events revealed that the FS-PC synaptic AR was much weaker than autaptic AR, by showing shorter duration and less AR events after FS neuron firing (Figure 3B–C).

**Figure 4:**
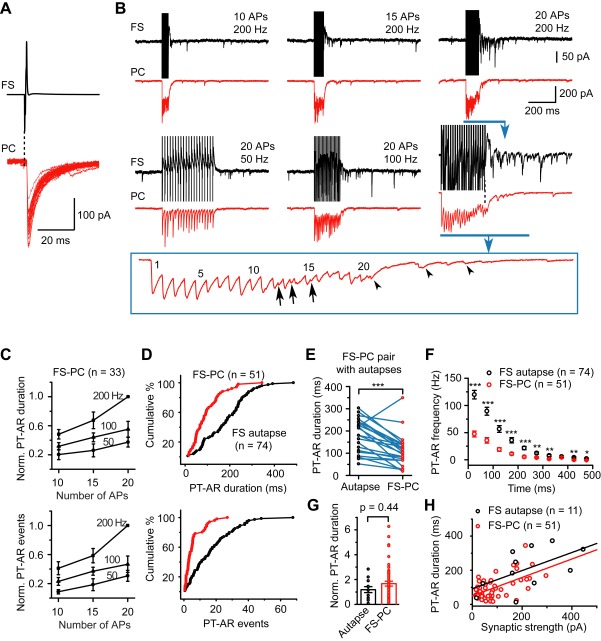
AR strength depended on the type of synaptic connections. (A) Overlay of unitary IPSCs in the postsynaptic PC evoked by single APs in the presynaptic FS neuron. (B) Example current traces (same FS-PC pair as in A) showing the dependence of AR strength at both autaptic and FS-PC synaptic connections on the stimulus intensity. Box, expanded trace for clarity. Note that AR could occur during (arrows) and after (arrowheads) the train of stimulation. (C) Group data showing the dependence of AR strength on the number and the frequency of presynaptic APs in FS-PC pairs (two-way ANOVA analysis, *p*<0.001). (D) Cumulative frequency distribution indicates that FS autapses had longer PT-AR duration and more events than FS-PC synapses (*p*<0.001 for both parameters). (E) Group data from FS-PC pairs (*n* = 22) that exhibited both autaptic and synaptic connections. (F) Group data showing that FS autapses had significantly higher PT-AR frequency than FS-PC synapses. (G) Bar plot of the normalized PT-AR duration (normalized to the average peak amplitude of unitary IPSCs). This normalization reveals the dependence of PT-AR duration on the synaptic strength. (H) Plot of the PT-AR duration as a function of synaptic strength. One outlier data point (688, 238) from autaptic connections has been excluded. Solid lines are linear fits (Black: r = 0.50, *p* = 0.07; red, r = 0.57, *p*<0.001). * *p*<0.05; ** *p*<0.01; *** *p*<0.001.

To further elucidate the differences between FS autaptic and FS-PC synaptic AR, we performed dual recordings in voltage-clamp mode. Single stimulation (0.3∼0.5-ms step command from −70 to 40 mV) evoked monosynaptic IPSCs in both FS neuron (Figure 1) and PC (Figure 4A). Among 343 FS-PC pairs tested, we found 79 FS-to-PC (23.0%) and 32 PC-to-FS (9.3%) connected pairs, and five (1.5%) bi-directionally connected pairs. FS-PC IPSCs had a failure rate of 0.03±0.03%, an average peak amplitude of 90.9±10.2 pA, an onset latency of 1.00±0.04 ms, rise time of 0.79±0.04 ms, and decay time constant of 8.5±0.7 ms (*n* = 51 pairs). As shown in Figure 4B–C, both autaptic and FS-PC synaptic AR show dependence on the number and frequency of FS neuron discharges (ANOVA, *p*<0.001). In sharp contrast to autapses, PT-AR at FS-PC synapses had shorter duration (98.8±9.6 versus 187±11 ms) and less IPSC events (6.1±0.8 versus 17.7±1.6, *n* = 51 FS-PC pairs and 74 FS neurons with autaptic connections) in response to presynaptic firing of 20 APs at 200 Hz. Cumulative frequency distribution of all these recordings revealed significant differences in the duration and total number of events of PT-AR between FS autapses and FS-PC synapses (K-S test, *p*<0.001 for both duration and events; Figure 4D). Consistently, in 22 FS-PC pairs that showed both autaptic and synaptic connections, the duration and total events of PT-AR at autapses were significantly larger than those of FS-PC synapses (*n* = 22, *p*<0.001, paired *t* test; Figure 4E). Examination of the PT-AR frequency revealed that FS autapses exhibited significantly higher frequency than FS-PC synapses (Figure 4F).

To examine whether the strength of PT-AR depends on the size of AP-triggered synaptic responses, we normalized the PT-AR duration by the average peak amplitude of unitary IPSCs (synaptic strength). We found that although PT-AR duration in autaptic connections was longer than that in FS-PC connections, the normalized PT-AR duration showed no significant difference between the two types of connections (Figure 4G), indicating a dependence of AR duration on synaptic strength. Consistently, PT-AR duration showed a positive linear correlation with the average IPSC amplitude, and the slopes were 0.53 and 0.54 ms/pA for FS autapses and FS-PC synapses, respectively (Figure 4H). Similar results were observed in the total number of PT-AR events. This analysis revealed the dependence of AR strength on the size of synchronous synaptic responses that may be attributed to the differential AR strength at different types of synapses.

We next analyzed the dependence of AR differences between FS autapses and FS-PC synapses on the clinical parameters of patients (sex, age, time since seizure onset, cause of disease, occurrence frequency, and seizure duration). We found no obvious dependence of PT-AR duration differences in the two types of connections on these parameters (Figure S1); that is, the duration at FS autapses was always significantly longer than that at FS-PC synapses if we categorized the patients by these parameters. Interestingly, we detected a small but significant difference in PT-AR duration at autaptic connections between patients with seizure duration of 1–3 min and those ≥3 min (Figure S1), suggesting a role of AR in regulating epileptic activities.

Together, these results demonstrated that, in human epileptic tissue, AR also occurred at FS-PC synaptic connections but was substantially weaker than that in FS autaptic connections.

### Ca^2+^ Dependence of AR in Human Cortex

**Figure 5:**
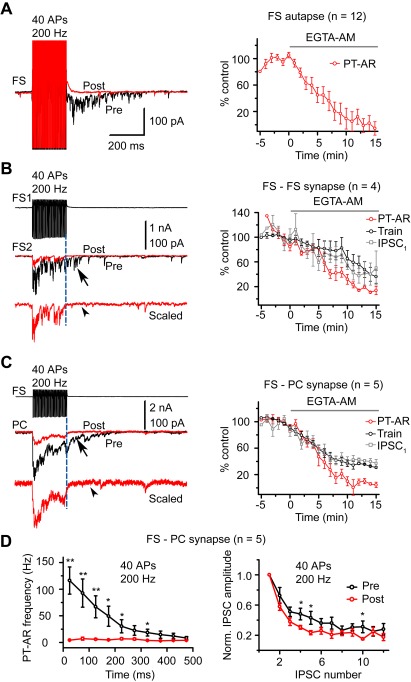
AR from human FS neurons was Ca^2+^ dependent. (A) Bath application of EGTA-AM (100 µM) could block PT-AR in FS autaptic connections. Left, example traces pre- (black) and post- (red) drug application. Right, group data from 12 FS neurons with autaptic connections. (B) EGTA-AM blocked AR in FS-FS synaptic connections. Left, FS1 stimulation (top) evoked barrages of IPSCs in FS2 (middle, black) that outlasted the train stimulation. Middle, EGTA-AM not only blocked the PT-AR (arrow) but also reduced the amplitude of IPSCs during the train (Train). Bottom, the red trace (post-EGTA-AM) shown in the middle was scaled to the peak of control trace to show the absence of PT-AR (arrowhead). Right, group data from 4 FS-FS pairs. IPSC_1_ indicates the peak amplitude of the first IPSC in the train. (C) EGTA-AM blocked PT-AR in FS-PC synaptic connections. Note the absence of PT-AR in the scaled trace (arrowhead). Right, group data from 5 FS-PC pairs. The number of quanta released during (Train) and after the train (PT-AR) was calculated using deconvolution analysis (see Materials and Methods). (D) Left, PT-AR frequency before (black) and after (red) EGTA-AM perfusion; right, short-term depression was accelerated after EGTA-AM application. Only the first 12 IPSCs were shown. * *p*<0.05; ** *p*<0.01.

Accumulation of Ca^2+^ in presynaptic terminals during trains of stimulation could be responsible for the occurrence of AR in hippocampus [32] and somatosensory cortex [33]; we therefore investigated the role of background or residual Ca^2+^ in the occurrence of AR in human neocortex. In the presence of EGTA-AM (100 µM), a membrane permeable Ca^2+^ chelator, FS autaptic, and FS-FS/FS-PC synaptic AR in human cortical tissues were completely blocked after 15-min drug application (Figure 5). The PT-AR in FS autapses was decreased to −5.6±10.2% of control (*t* test, *p*<0.001, *n* = 12, Figure 5A). In FS-FS synapses, EGTA-AM blocked the release after the train (PT-AR) and reduced the release during the train (Train) as well as the peak amplitude of the first IPSC (IPSC_1_) in the train (PT-AR: 13.8±6.8%, *p* = 0.003; Train: 36.1±10.7%, *p* = 0.005; IPSC_1_: 50.2±27.4%, *p* = 0.08, *n* = 4; Figure 5B). We observed similar results in FS-PC synapses; PT-AR, Train, and IPSC_1_ were decreased to 4.4±4.1%, 31.0±1.3%, and 37.1±5.6% of control, respectively (*p*<0.001 for all comparisons, *n* = 5, Figure 5C). As shown in Figure 5C–D, PT-AR events in FS-PC pairs were completely abolished by the application of EGTA-AM. The blockade of PT-AR events was accompanied by an increase in short-term depression of the synchronous IPSCs in response to 40 APs at 200 Hz (Figure 5D), consistent with the finding that EGTA sharpens the initial decay phase of calcium transient [37].

Further recordings showed that the replacement of normal ACSF with Ca^2+^-free ACSF completely blocked the occurrence of IPSCs, including the PT-AR in FS-PC synapses (unpublished data), suggesting that AR requires Ca^2+^ entry through Ca^2+^ channels. Together, these results indicate that AR was dependent on the buildup of intracellular Ca^2+^ during the train of stimulation.

### Comparison of AR between Non-Epileptic and Epileptic Human Tissues

Previous studies demonstrated that alteration of GABAergic inhibition could contribute to epileptogenesis. We speculated that the asynchronous GABA release from FS output synapses may undergo changes in the epileptic brain tissue. Furthermore, the findings in epileptic cortical tissues described above may be due to lengthy treatments of patients with anti-epileptic drugs. We thus performed recordings from FS neurons of surgically removed non-epileptic peri-tumor tissues and compared the AR properties with those of epileptic tissues.

**Figure 6:**
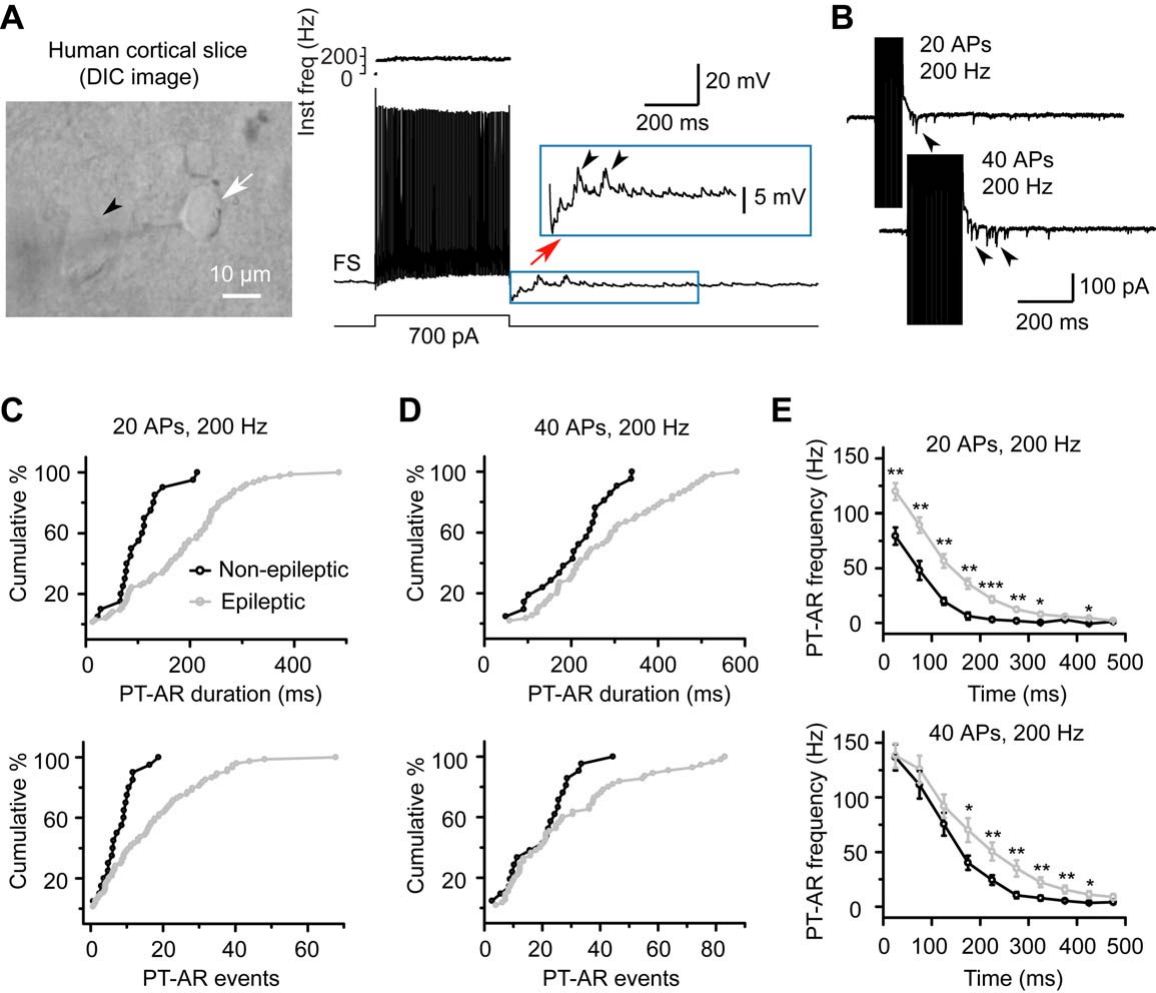
Difference in AR strengths between non-epileptic and epileptic human tissue. (A) Left, a DIC image of the recorded FS neuron (arrow) and neighboring cells in a slice obtained from non-epileptic human tissue. Arrowhead indicates a pyramidal neuron. Right, example recording shows that AR events occurred after a train of APs evoked by step current injection. (B) PT-AR duration and total number of events depended on the number of APs (voltage clamp). Same cell as in (A). (C) Cumulative distribution of the PT-AR duration (top) and events (bottom) from the tested FS neurons with autaptic connections (20 APs at 200 Hz). *n* = 20 and 74 for non-epileptic and epileptic tissues, respectively. (D) Similar plot as in (C) except for 40 APs (*n* = 21 and 55, respectively). (E) Plot of the PT-AR frequency (bin size: 50 ms) as a function of time since the stop of the train stimulation. Note that PT-AR frequency in the epileptic tissue (gray) was significantly higher than that in the non-epileptic tissue (black). * *p*<0.05; ** *p*<0.01; *** *p*<0.001.

Similar to the epileptic tissue, the autaptic AR occurred after a train of APs evoked by current injections (Figure 6A) or voltage steps (Figure 6B) in FS neurons, and the strength of AR depended on the stimulation intensity (Figure 6B). However, the average PT-AR duration (103±11 ms) and total events (8.1±1.0) of autaptic AR (20 APs at 200 Hz, *n* = 20) were significantly lower than the values found in the epileptic tissue (187±11 ms and 17.7±1.6 events, *n* = 74; K-S test, *p*<0.001 for both parameters; Figure 6C). Significant differences in AR properties were also observed when FS neurons fired 40 APs at 200 Hz (K-S test, *p*<0.05, Figure 6D). In addition, the non-epileptic tissue had lower PT-AR frequencies than the epileptic tissue (Figure 6E). Thus, asynchronous GABA release at FS autaptic connections is elevated in human epileptic patients.

### Enhancement of AR in a Rat Model of Epilepsy

**Figure 7:**
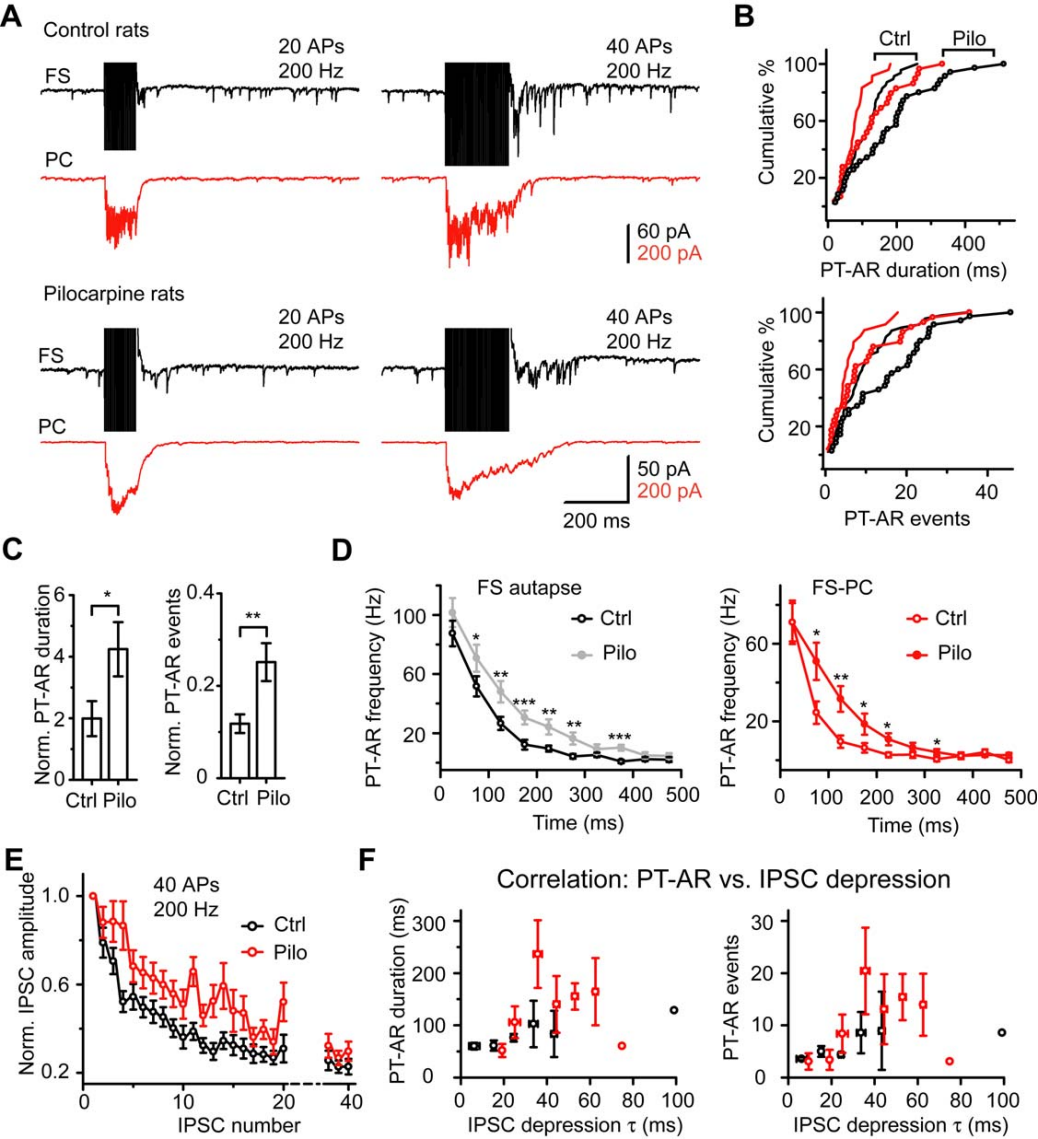
Enhancement of AR in the pilocarpine model of epilepsy. (A) Example FS-PC pair recordings in control and pilocarpine-treated rats. (B) Cumulative frequency distribution of the tested FS autaptic and FS-PC synaptic connections by the PT-AR duration (top) and PT-AR events (bottom). Control (Ctrl): *n* = 47 FS autaptic connections (black) and 25 FS-PC synaptic connections (red); Pilocarpine (Pilo): *n* = 35 and 32, respectively. (C) Bar plot of the PT-AR duration and events normalized to the average peak amplitude of unitary FS-PC IPSCs. *p* = 0.02 and *p* = 0.005, respectively. (D) Plot of the PT-AR frequency (bin size: 50 ms) as a function of time since the stop of the train stimulation. Left, FS autaptic connections; right, FS-PC synaptic connections. Note that PT-AR frequency in pilocarpine rats was significantly higher than that in control rats. (E) Normalized IPSCs during the train stimulation (40 APs at 200 Hz) in control and pilocarpine rats (*n* = 23 and 25 pairs for control and pilocarpine rats, respectively). (F) Correlation between PT-AR properties (duration and events) and the time course of IPSC depression (τ). The τ value was calculated by an exponential fit of the normalized IPSCs as shown in (E). Note that PT-AR duration and number of events increase with increasing depression time course. Two outlier data were not binned.

Next, we sought to examine whether or not there are differences in AR between FS autapses and FS-PC synapses in cortical slices obtained from adult rats and whether AR is subjected to change after the induction of epileptic seizures (Figure 7). In these experiments, we employed the pilocarpine model of status epilepsy (see Materials and Methods) that mimics human temporal-lobe epilepsy [38],[39]. Recordings from FS neurons or FS-PC pairs in control and pilocarpine-treated rats showed that the connectivity probabilities from FS to neighboring PCs (<50 µm apart) were 33.7% (*n* = 29/86) and 37.3% (*n* = 38/102) for control and pilocarpine-treated rats, respectively, much higher than those in the opposite directions (PC-to-FS, 3.5% for control, *n* = 3/86; 1.0% for model animals, *n* = 1/102).

Consistent with previous reports [32],[33], we found that, in prefrontal cortical slices from control rats, the strength of AR in FS autapses was dependent on the intensity of stimulation. In response to 20 APs at 200 Hz, PT-AR had an average duration of 53.9±4.2 ms and an average number of 3.3±0.4 events (*n* = 47). These values increased to 110±9 ms and 9.7±1.1 events, respectively, when the number of APs was increased to 40 (Figure 7A). Similar results were observed in FS-PC synaptic connections (20 APs: 36.8±4.4 ms and 1.8±0.3 events; 40 APs: 79.5±8.5 ms and 5.9±0.9 events; *n* = 25 pairs; Figure 7A). Again, cumulative frequency distribution of the recordings by the PT-AR duration and events showed that FS autapses exhibited significantly stronger PT-AR than FS-PC synapses (K-S test, *p*<0.001 and *p*<0.05 for duration and events, respectively; *n* = 47 FS neurons with autapses and 25 FS-PC pairs; Figure 7A–B). Together with the findings obtained from human tissue, these results indicate that the dependence of AR strength on the type of synaptic connections is a fundamental property of FS neuron output synapses.

Because postsynaptic spiking may cause an elevation of intracellular Ca^2+^ and send retrograde signals to presynaptic terminals to regulate synaptic transmission [40], we then investigated whether the AR strength was dependent on postsynaptic spiking. In FS-PC pairs, the PT-AR duration and number of events showed no substantial changes after paired stimulations in the presynaptic FS neuron and the postsynaptic PC (40 APs at 200 Hz in both cells simultaneously; Figure S2). The PT-AR duration was 76.4±13.4 and 76.2±13.5 ms (paired *t* test, *p* = 0.98), and the total number of PT-AR events was 5.6±1.4 and 5.0±1.2 for control (firing in FS neuron only) and paired firing (*p* = 0.36, *n* = 6 FS-PC pairs), respectively. This result indicates that postsynaptic spiking is not required for the occurrence of AR from presynaptic FS neurons.

Next, we analyzed the strength of AR and synchronous release in pilocarpine-treated rats. Similar to control rats, we also observed that the strength of PT-AR was different between FS autapses and FS-PC synapses, with stronger PT-AR detected at FS autapses (Figure 7A–B). No significant difference in the peak amplitude of single AP-evoked unitary IPSCs was observed (78.9±16.3 pA in control versus 62.0±12.9 pA in model rats, *n* = 25 and 32, respectively; *t* test, *p* = 0.07) in FS-PC pairs, indicating that the basic neurotransmission was unchanged in this model of epilepsy.

Importantly, we found that AR at both FS autaptic and FS-PC synaptic connections were significantly increased in pilocarpine-treated rats (Figure 7B). In response to 40 APs at 200 Hz, the average duration and number of events were increased to 180±21 ms and 15.4±1.9 events in FS autaptic connections (K-S test, *p* = 0.002 and *p* = 0.005, *n* = 35) and to 126±16 ms and 9.6±1.7 events in FS-PC synapses (*p* = 0.015 and *p* = 0.066, *n* = 32). Cumulative frequency distributions of recordings obtained from epileptic animals by PT-AR duration and events showed a rightward shift in comparison with those from control rats (Figure 7B). We next normalized the duration and number of events to the peak amplitude of unitary IPSCs (synaptic strength); this normalization also showed a significant PT-AR increase in pilocarpine-treated rats (*t* test, *p* = 0.02 and *p* = 0.005, respectively; Figure 7C). The mean frequencies of PT-AR events in both FS autaptic and FS-PC synaptic connections were also significantly larger in epileptic animals (Figure 7D). Further analysis on the ratio of PT-AR to total release (PT-AR/Total, Figure S3) revealed a significant increase from 3.8±0.5% (control rats, *n* = 24 FS-PC pairs) to 7.6±0.9% (pilocarpine rats, *n* = 32 pairs; *p*<0.001) for FS neurons firing 20 APs at 200 Hz. Similar results were obtained when FS neurons fired 40 APs (Figure S3).

A recent study demonstrated that AR occurred in response to high-frequency second-long stimulation of the presynaptic FS interneuron [33]; we therefore examined whether there is a difference between the FS autaptic and FS-PC synaptic AR after these strong stimulations. In human neocortical slices (Figure S4), firing of 300 APs at 150 Hz in FS neuron caused long-lasting AR in both FS autapses (*n* = 18 FS neurons) and FS-PC synapses (*n* = 10 pairs). Consistent with the results described above (Figure 4F), we also observed differences of AR frequency in the two types of connections. Again, comparison between control and pilocarpine-treated rats showed that AR frequency in both FS autapses and FS-PC synapses was significantly increased in epileptic animals (Figure S4).

Although the basal transmission exhibited no significant change in model animals, the short-term depression of synchronous release at FS-PC synapses in response to stimulation of 40 APs at 200 Hz (Figure 7E) were significantly reduced in model animals. Further analysis revealed a close correlation between PT-AR strength and the time course τ of IPSC depression; the PT-AR duration and events showed an increase with increasing time course (Figure 7F). Short-term plasticity is tightly coupled to the concentration of presynaptic Ca^2+^ [41]; reducing residual Ca^2+^ levels could significantly accelerate short-term depression (Figure 5D, also see [42]). The reduction of short-term depression may therefore reflect an elevation of residual Ca^2+^ concentration at the presynaptic axon terminals, in line with the enhancement of AR in model animals.

Together, these results obtained from rats indicate that the strength of AR at FS neuron axon terminals was elevated in epileptic neocortical tissue, possibly resulting from an increase in residual Ca^2+^ levels.

### Role of AP Waveform Changes in Mediating AR Enhancement

**Figure 8:**
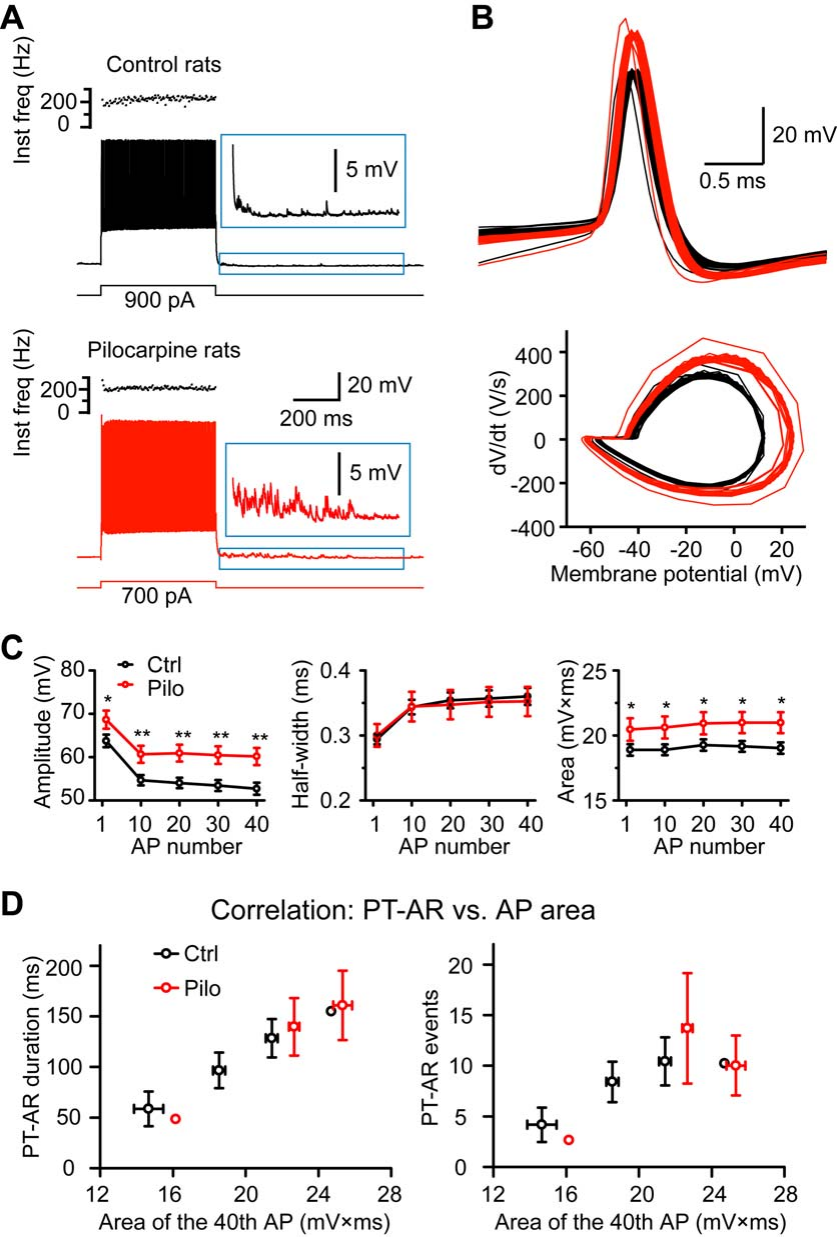
Changes in AP waveform of FS neurons in rat epileptic tissues. (A) Example firing pattern of FS neurons with autaptic AR in control and pilocarpine rats. Insets, expanded traces showing the occurrence of autaptic AR. (B) Overlay of the first 40 APs and their phase plots. Note the differences in AP peak amplitudes. (C) The average amplitude, half-width, and integrated area of the 1^st^, 10^th^, 20^th^, 30^th^, and 40^th^ AP (Ctrl: *n* = 33; Pilo: *n* = 21). Note the significant increase in peak amplitude and integrated area (but not the half-width) of APs in model animals. (D) Correlation of PT-AR duration (left) and events (right) in FS autapses (Ctrl: *n* = 25; Pilo: *n* = 13) with the integrated area of APs. Note that PT-AR duration and events increases with larger AP area. Two outlier data were not binned.

Changes in AP waveforms can regulate Ca^2+^ entry during APs [43], so we next investigated whether AP waveforms of FS neurons were altered in model animals (Figure 8). We analyzed APs (fired at ∼200 Hz) evoked by 500-ms current steps and found that although the AP threshold (1^st^ AP: −46.0±0.7 mV in control versus −45.3±1.3 mV in pilocarpine rats, *p* = 0.7) and the half width showed no significant difference, all APs during the train in pilocarpine rats exhibited larger peak amplitude and integrated area than those in control rats (Figure 8A–C). The average peak amplitudes of the first and the 40^th^ AP were 68.6±2.1 and 60.2±2.0 mV, respectively, in model animals, significantly higher than those in control animals (63.7±1.4 and 52.7±1.4 mV, *t* test, *p*<0.05 for the first AP, *p*<0.01 for the 40^th^ AP and other APs). Further analysis revealed that AP waveform changes correlated well with the AR strength; PT-AR duration and events increases with increasing AP amplitude and area (Figure 8D).

**Figure 9:**
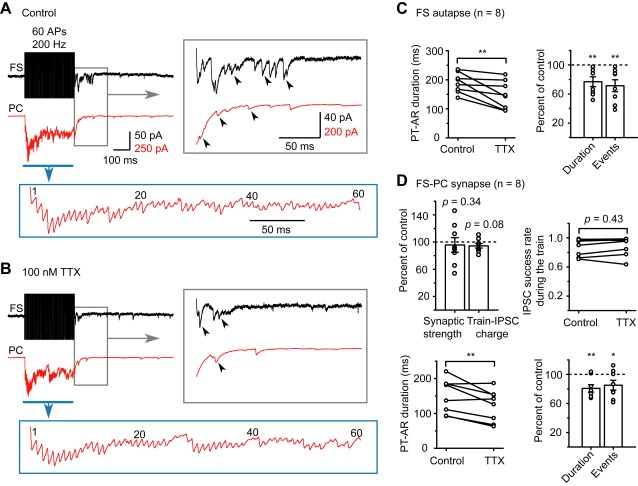
AR strength could be reduced by the bath application of a low concentration of TTX. (A) Example recording from epileptic rat tissue showing the occurrence of FS autaptic and FS-PC synaptic AR before the application of TTX. Parts of the traces were expanded for clarity. (B) During the period from 120 to 180 s following the onset of 100 nM TTX application, the autaptic and synaptic AR were substantially reduced. Note that the synchronous release during the train stimulation was largely preserved. Same cell as in (A). (C) Group data from FS neurons with autaptic connections showing that both the PT-AR duration and total number of events were significantly reduced in the presence of TTX (100 nM). (D) In FS-PC pairs, TTX had no significant effects on the synaptic strength (the average peak amplitude of the first IPSC), the success rate, and the integrated charge of IPSCs evoked during the train stimulation, but significantly reduced the PT-AR duration and total number of events. * *p*<0.05; ** *p*<0.01.

To investigate the role of the AP amplitude increase in mediating the enhanced AR, we reduced the AP amplitude by an amount similar to that of AP increase in epileptic animals, with the treatment of a low concentration of TTX (Figure 9, also see Figure S5). During the period from 120 to 180 s following the onset of TTX treatment (100 nM), we found a slight change in the threshold and half width (Figure S5) but a significant reduction in the average peak amplitude of APs in FS neurons (from 75.4±1.9 to 67.4±2.9 mV, *p* = 0.001, *n* = 9). Although we observed no significant TTX effect on the synaptic strength (average amplitude of the first IPSCs), the success rate, and the total integrated charge of IPSCs during the train stimulation (40 or 60 APs at 200 Hz) in FS-PC pairs (Figure 9A,B, and D), the PT-AR duration and total number of events significantly decreased to 76.9±6.7% and 71.4±8.2% of control in FS autaptic connections (*p*<0.01 for both parameters, *n* = 8; Figure 9C) and to 80.7±5.1% and 85.1±6.7% of control in FS-PC synaptic connections (*p*<0.01 and 0.05, *n* = 8; Figure 9D), respectively.

Together, our results demonstrate that AR occurs in all GABAergic synapses of FS neurons, with autapses having the strongest AR, and the generation of epileptic seizures correlates with an increase in AR at both FS autapses and FS-PC synapses, suggesting that AR is subjected to modification during elevated network activities and is involved in regulating epileptic activities.

## Discussion

In this study, we show that asynchronous GABA release occurs at the output synapses of FS neurons, including FS autapses and FS-FS and FS-PC synapses in the human neocortex. Interestingly, AR at FS autapses is the strongest among all these synaptic connections. This differential AR may be attributed to the differences in the synaptic strength. In comparison with the non-epileptic human tissue, AR strength was significantly stronger in the epileptic tissue. We further demonstrate the existence of AR differences at FS autapses and FS-PC synapses in rats, and AR strength is substantially enhanced in the pilocarpine model of epilepsy, possibly resulting from an increase in peak amplitude of FS neuron APs and elevation of residual Ca^2+^. Consistent with previous reports [32], AR from FS neurons in human cortical tissue is dependent of the background or residual Ca^2+^ in the presynaptic terminals but independent of postsynaptic spiking. Together, these results, to our knowledge, provide the first piece of evidence showing the occurrence of GABAergic AR in human tissue, and importantly reveal an elevation in AR in the epileptic neocortex, suggesting a role of AR from FS neurons in regulating the synchrony of network activities and thus shaping epileptiform activities.

### AR Is a Fundamental Property of Neocortical FS Neuron

Asynchronous GABA release was originally reported at output synapses of hippocampal cholecystokinin-containing interneurons onto granule cells in the dentate gyrus [32]. Unlike these cholecystokinin neurons, parvalbumin-containing FS interneurons release GABA in a tightly synchronized manner in response to single or a burst of APs. However, a recent study reported the occurrence of AR at output synapses of FS neurons onto themselves (autapses) and PCs in the rat somatosensory cortex [33]. Using human neocortical tissue, we demonstrate that AR occurs at all synapses of FS neurons in human neocortex, including synaptic contacts onto other FS neurons. In addition, the results show a dependence of AR strength on the type of connections, and FS autapses exhibit much stronger AR than FS-FS and FS-PC synapses. These findings were obtained from human epileptic tissue and thus may reflect synaptic modifications after epileptic seizures. However, similar results were observed in normal adult rats, indicating that AR is a fundamental property of neocortical FS neurons across different species and that the differences in AR strength between different types of connections are not associated with epilepsy.

Why do FS autapses have the strongest AR than other synapses? One possibility is that the AR strength depends on the size of synaptic responses. Indeed, our results showed a correlation between the amplitude of unitary IPSCs and the duration of PT-AR; no significant difference was observed if the duration was normalized to the average peak amplitude of unitary IPSCs (Figure 4G–H). Another potential mechanism may lie on retrograde signals. At the autaptic connection, firing of the FS neuron may cause an increase in intracellular Ca^2+^ and consequently send retrograde signals to presynaptic terminals, such as nitric oxide, BDNF, and GABA [40],[44]. AP burst-induced GABA release from the dendrites unlikely contributes to the elevated spontaneous release because 307/392 FS neurons tested in this study showed no AR following high-frequency discharges. Our experiments in FS-PC pairs showed no changes in PT-AR duration and number of sIPSC events after pairing postsynaptic and presynaptic firing (Figure S2), indicating that postsynaptic spiking has no effect on presynaptic asynchronous GABA release.

### Role of Background Ca^2+^ in AR

Previous findings [32] together with our results (Figure 5) demonstrate that the slow Ca^2+^ buffer EGTA can efficiently block the AR, indicating the dependence of the residual Ca^2+^. One distinct characteristic of FS interneurons is their expression of parvalbumin [12]; this Ca^2+^-binding protein functions as a slow Ca^2+^ buffer in presynaptic terminals and participates in short-term plasticity of synaptic transmission [45]–[48]. The presence of parvalbumin may prevent the occurrence of AR in FS neuron terminals. In hippocampal dentate gyrus, Hefft and Jonas [32] reported that AR at output synapses of parvalbumin-containing interneurons is almost absent (also see [49],[50]). In the neocortex, Manseau et al. [33] reported recently that parvalbumin-expressing interneurons also have strong AR but are weaker than those that lack of parvalbumin, indicating an important role of parvalbumin in controlling the strength of AR. Regulation of parvalbumin expression by neuronal activity may contribute to changes in AR strength in epileptic brain tissue. Comparison of short-term depression of synchronous IPSCs (Figure 7E–F) suggests that the kinetics of presynaptic Ca^2+^ may be altered in pilocarpine model animals, and this alteration could lead to the enhancement of AR. Interestingly, our results demonstrate that the peak amplitude of APs in FS neurons was substantially increased in the epileptic tissue, possibly resulting from homeostatic regulation of neuronal intrinsic properties (e.g., Na^+^ channel properties) [51],[52]. This AP-waveform change may cause more Ca^2+^ entry during APs [43] and consequently increase the total residual Ca^2+^ after a train of stimulation. Indeed, when we perfused the slice with TTX at a low concentration that showed no effect on the synchronous synaptic transmission but slightly decreased the amplitude of APs, PT-AR duration and number of events were significantly reduced (Figure 9). These findings suggest that changes in AP waveforms may play an important role in regulating AR strengths.

The distance between the Ca^2+^ source (voltage-gated Ca^2+^ channels) and the sensor of exocytosis may differ at different types of synaptic connections and thus determine the occurrence and the strength of AR. At the hippocampal cholecystokinin interneuron terminals, this distance is large, leading to long-lasting intracellular Ca^2+^ transient and thus asynchronous vesicle release; whereas at parvalbumin interneuron terminals, the Ca^2+^ channels locate closely with sensors, allowing fast and precise synchronous release of vesicles [32],[53]. Our results demonstrate that, in human neocortical slices, bath application of EGTA-AM not only completely blocked the AR but also substantially reduced the synchronous release during the train stimulation, suggesting a large diffusional distance between the Ca^2+^ source and the sensor of exocytosis in FS neuron terminals [32]. Whether this distance is subjected to modulation by cortical activities and whether different Ca^2+^ sensors [54],[55] are involved in regulating AR at FS neuron terminals remain to be further examined.

Changes in AR could also be explained by the homeostatic regulation of synaptic strength and intrinsic neuronal property in response to the epileptic activity. Previous studies have shown that changes in network activities can lead to alterations of synaptic strength in both excitatory and inhibitory synapses, leading to adjustment of the firing rate of individual neurons within a physiological range [56]. For example, a chronic elevation of network activity in cultured neurons decreased excitatory but increased inhibitory synaptic strengths of input synapses in PCs [57],[58], suggesting that the homeostatic synaptic scaling could help to maintain a balance between cortical excitation and inhibition. The enhanced asynchronous GABA release in both human and rat epileptic tissues may thus reflect a homeostatic change in GABAergic inhibition that can counterbalance the excessive excitation following prolonged epileptic activities. Considering the inhibitory nature of GABA and the desynchronizing effect of AR [59], we speculated that the enhanced AR in epileptic tissue might be anti-epileptic. In addition to homeostatic changes in synaptic strength, alteration in intrinsic neuronal properties could also occur when the level of network activity changes [59]. The density or the composition of ion channels is finely regulated by neuronal activities. In animals that developed status epilepsy, previous studies [60],[61] revealed a down-regulation of ion channels that mediate the dendritic A- and h-currents, resulting in changes in firing patterns and synaptic integration in hippocampal and cortical PCs. In this study, we found an increase in the peak amplitude of APs in FS neurons in pilocarpine-treated rats (Figure 8), suggesting an upregulation of Na^+^ channel density in these neurons, consistent with homeostatic regulation in response to epileptic activities.

### Comparison of AR in Non-Epileptic and Epileptic Tissues

Because it is not possible to obtain normal brain tissue from healthy humans, we used the discarded peri-tumor tissues from patients with brain tumors who exhibited no clinical symptoms of epileptic seizures. Recordings from these non-epileptic tissues were considered as control for the effects of epileptic activities or lengthy treatment of anti-epileptic drugs. Our observation of AR occurrence in both control and epileptic human tissues indicated that AR is a fundamental property of human neocortical FS neurons and not due to drug treatment. Furthermore, our results clearly show that AR at FS autaptic connections in the epileptic tissue was substantially stronger than that in the non-epileptic tissue (Figure 6). Similar results were obtained from experiments using the pilocarpine rat model of epilepsy. In addition to FS autapses, enhanced AR was also observed in FS-PC synaptic connections in epileptic rats. These findings also indicated that the pilocarpine model of epilepsy is an appropriate model for temporal lobe epilepsy. Similar AR enhancement in pilocarpine-treated rats also supports the notion that the enhanced AR in human epileptic patients was not due to the treatment of anti-epileptic drugs.

Further analysis on clinical parameters (Figure S1) revealed no dependency of the differences in AR duration between FS autaptic and FS-PC synaptic connections on patients' sex, age, time since the seizure onset, causes of seizure, occurrence frequency, and duration of seizure. Only those patients with relatively long-lasting seizures (i.e., seizure duration longer than 3 min) had significantly longer AR duration at autaptic connections (Figure S1), indicating a role of the enhanced autaptic AR in regulating epileptic seizures. Together, our results revealed an alteration of AR-induced long-lasting self-inhibition in FS neurons and inhibition in PCs, which may contribute to the generation and maintenance of the epileptiform activity.

### Physiological Significance

Our results demonstrate that AR in FS neuronal terminals occurs not only in rat but also in human neocortex, indicating that AR is a fundamental property of the cerebral cortex and participates in cortical functions. In a normal brain, asynchronous GABA release after high-frequency firing provides long-lasting inhibition and enables gain control of the postsynaptic neurons [32],[62]. Moreover, a recent study [33] revealed that asynchronous GABA release from FS neurons causes reduction of the discharge reliability and precision in postsynaptic neurons, particularly in PCs, a mechanism that may cause desynchronization of cortical activities. Therefore, on the one hand, FS neurons synchronize large populations of neurons during various cortical oscillations through their synchronous release at relatively low firing rates; on the other hand, they desynchronize neuronal networks through their AR when excessive excitation arises. The later process may play an important role in preventing runaway excitation and diminishing the generation and propagation of epileptiform activities. Consistently, our experiments in human and rat epileptic tissue revealed an increase in asynchronous GABA release at both autaptic and FS-PC synaptic connections, leading to desynchronization of FS/PC activities and the regulation of the generation and maintenance of epileptiform activities. These results are also in line with a recent report showing that neuronal firing during seizure initiation and propagation in epileptic human patients was highly heterogeneous rather than hypersynchronous [63].

Previous studies mainly focused on the alteration of basic neurotransmitter release at GABAergic synapses in epileptic brain tissue. Given that cortical neurons encounter large depolarizations and high-frequency discharges during epileptiform activities, it is important to investigate the changes in AR evoked by high-frequency discharges. Interestingly, we found no significant difference in the amplitude of unitary IPSCs between control and epileptic animals. With high-frequency stimulation of FS neurons, we did observe an increase in asynchronous GABA release in the rat model as well as in human epileptic tissues. Therefore, our results suggest that asynchronous GABA release in FS interneurons could be a target for the development of novel anti-epilepsy drugs.

## Materials and Methods

### Ethics Statement

The protocols for handling and using the human brain tissue had been approved by the Biomedical Research Ethics Committee of Shanghai Institutes for Biological Sciences (License No. ER-SIBS-221004). The use and care of animals complied with the guidelines of the Animal Advisory Committee at the Shanghai Institutes for Biological Sciences.

### Human Patients and Pilocarpine-Treated Epilepsy Model

Human neocortical slices were prepared from brain tissues that had to be removed surgically to cure intractable epileptic seizure and brain tumor. Prior to the surgery, all patients and relatives or their legal representatives had provided written informed consent. Brain tissues from 52 human patients with frontal or temporal lobe epilepsy (aged from 5 to 42 y) and two patients with brain tumors (57 and 69 y), who showed no clinical symptoms of epileptic seizures, were used in this study. A small block of the discarded tissue was immediately immersed into an ice-cold oxygenated (0°C, 95% O_2_ and 5% CO_2_) sucrose solution (modified artificial cerebrospinal fluid, ACSF) in which the NaCl was substituted with equiosmolar sucrose and dextrose was reduced to 10 mM. We then sliced the brain tissue in this sucrose solution. In the case of brain tumor, we only used the peri-tumor tissue that had clear cortical layers (from layer 1 to 6) and white matter.

Rat neocortical slices were obtained from adult Sprague-Dawley (SD) rats and pilocarpine-treated epileptic rats. A classic protocol was utilized to produce a pilocarpine model of epilepsy [38],[39]. Thirty minutes prior to pilocarpine injection, cholinergic antagonist methyl scopolamine nitrate (2 mg/kg, Sigma) was administered to minimize the peripheral effects of pilocarpine. The animals (220–250 g in weight) were then randomly divided into two groups: the pilocarpine group, which received a single injection of pilocarpine hydrochloride (345 mg/kg, Sigma), and the control group, with an injection of normal saline. All rats in the pilocarpine groups showed severe epileptic behavior ∼15 min after the injection, and this convulsive behavior was characterized by tonic-clonic generalized seizures (stage 5). The seizure intensity was evaluated according to Racine's criteria [64]. For every pilocarpine-treated rat, seizures were allowed to continue for 1.5 h and then terminated by the administration of diazepam (10 mg/kg, Sigma). Control rats also received the same dose of diazepam. Rats were then marked and sent back to their home cage. All drugs were applied through intraperitoneal injection. Seven days after the drug treatments, the occurrence of spontaneous epileptic seizures in these rats were monitored with a video-monitoring system. Rats with severe sustained seizures (showing clonic convulsions lasting for ∼20 s at least twice within 48 h) were selected for electrophysiological recordings, which were carried out 13–16 d after the pilocarpine injection. In our experimental condition, about half of the pilocarpine-treated rats showed spontaneous epileptic seizures within the monitoring time window. Animals were sent back to their home cages without video-monitoring until use. It is possible that spontaneous seizures might have occurred shortly before the preparation of slices.

### Electrophysiological Recording

Neocortical tissues from human patients, control rats, and pilocarpine-treated rats were utilized for patch clamp recordings. Slices with a thickness of 350 µm were cut in the ice-cold sucrose ACSF from a block of brain tissue with a vibratome (VT–1000S, Leica). After slicing, the slices were immediately transferred to an incubation beaker and incubated at 35.5°C for about 1 h and then room temperature until use. Recordings were performed in a submerged-style chamber at 36°C mounted under an infrared-differential interference contrast (IR-DIC) microscope (BX–51 WI, Olympus). The ACSF contained in mM: 126 NaCl, 2.5 KCl, 2 MgSO_4_, 2 CaCl_2_, 26 NaHCO_3_, 1.25 NaH_2_PO_4_, and 25 dextrose (315 mOsm, pH 7.4). Whole-cell recordings were achieved using a Multiclamp 700B amplifier (Molecular Devices). Signals were filtered at 10 kHz and then sampled by Micro 1401 mkII (Cambridge Electronic Design, Cambridge, UK) at 20 kHz using Spike 2 acquisition software. The impedance of patch pipettes was 5–7 MΩ with an internal solution containing in mM: 71 KCl, 72 Kgluconate, 2 MgCl_2_, 10 HEPES, 0.025 BAPTA, and 2 Na_2_ATP (288 mOsm, pH 7.2 with KOH). The reversal potential of Cl^−^ was −15 mV. Alexa Fluor 488 (50 µM) and biocytin (0.2%) were added to the internal solution for visualizing the morphology of recorded neurons. AP trains with varying AP number and frequency were evoked by step current injection (in current-clamp mode) or trains of voltage commands (voltage-clamp mode). Liquid junction potential (9 mV) has not been corrected for the membrane potential shown in the text and figures.

PCs and FS neurons were identified by their morphology and firing properties. PCs had pyramid-shaped soma and a single thick apical dendrite, exhibiting a regular firing pattern with adapting APs in response to a steady depolarization, whereas FS interneurons had a non-pyramidal soma and multiple primary dendrites and fired APs at very high frequencies (up to 500 Hz) without adaptation. After recording, the neurons were further identified using DAB-staining.

Picrotoxin (PTX, GABA_A_ receptor antagonist, Tocris), DL-2-Amino-5-phosphonopentanoic acid (APV, NMDA receptor antagonist, Tocris) and 6-Cyano-7-nitroquinoxaline-2,3-dione (CNQX, AMPA, and kainate receptor antagonist, Sigma), and EGTA-AM (membrane permeable calcium chelator, Invitrogen) were applied through bath perfusion.

### Data Analysis

We performed data analysis using Spike 2 and MATLAB (MathWorks, Bethesda, MD). The current trace of the postsynaptic neuron was transformed to slope for event detection. Individual AR events were detected by setting a slope threshold. The frequencies of spontaneous IPSCs during the 4-s period before the train stimuli were defined as the baseline frequency. PT-AR frequency was calculated with a bin size of 50 ms. The end of AR was defined as the time point of the last IPSC before AR frequencies reached the baseline frequency. The PT-AR duration was the time between the cessation of the train stimulation and the end of AR. To investigate the strength of asynchronous GABA release from FS neurons, we only measured the duration and number of spontaneous IPSC events after the train stimulation because it was difficult to detect the asynchronous events that occurred during the train, due to the mixture of synchronous and asynchronous release in response to presynaptic high-frequency discharges. For single AP-evoked unitary IPSCs, we obtained the peak amplitude by taking the differences between the peak and the baseline current. The rise time of unitary IPSCs was measured as the time from 10% to 90% of the peak amplitude; the decay time constant was obtained by fitting the decay phase with a single exponential function. The threshold of an action potential (AP) was defined as the membrane potential when dV/dt reached 20 V/s. The peak amplitude of an AP was measured as the voltage difference between the peak and the threshold. AP area was the integrated area above the level of AP threshold (Figure 8).

In order to calculate the quanta number during and after the train stimulation, we performed deconvolution analysis as described in previous studies [32],[49]. We first chose an isolated sIPSC from the barrages of sIPSCs that occurred after the train stimulation as a template. Then we used a template fit algorithm to detect the putative quantal IPSCs, within which we considered the peak amplitude of the smallest IPSC as the quantal size (5–10 pA). We fitted the rising phase of the previous IPSC template with a linear function and the decay phase with single exponential function and then scaled the amplitude to the quantal size to create an artificial quantal IPSC (IPSC_quantal_), which was used for deconvolution of the postsynaptic currents. We then performed the deconvolution with the following equation: Release rate = F^−1^[F (IPSC)/F(IPSC_quantal_)], where F is the discrete Fourier transform [32],[49]. The resultant trace was filtered by 5–10 repetitions of Gaussian-window FIR filter depending on the signal-to-noise ratio. For FS autapses, only the post-train spontaneous IPSCs were used for deconvolution, as action currents occurred during the train stimulation may cause errors in the following analysis. The quanta released after the train (PT-AR) was calculated as the integrated area of the post-train release rate. For FS-FS and FS-PC synapses, the amount of release during (Train) and after the train (PT-AR) were measured as the corresponding area of the release rate. The baseline release was the average during the 4-s period preceding the onset of the train stimulation. This baseline release was subtracted from the Train release and PT-AR. PT-AR ratio was the ratio of PT-AR to the total release (the sum of Train release and PT-AR; Figure S3).

Values were given as mean ± s.e.m., and error bars in figures also indicate the s.e.m. Significance of differences was assessed by two sample Student's *t* test. Two-sample Kolmogorov-Smirnov (K-S) test was performed if the data were not normally distributed. In order to study the intensity-dependent properties of synchronous release and PT-AR, two-way ANOVA analysis was utilized to test whether the number or frequency of APs in FS neurons had an effect on PT-AR.

## Supporting Information

Figure S1 Correlation between PT-AR duration and clinical parameters. (A) Significant difference in AR duration between autaptic and FS-PC connections was observed in both male and female patients (K-S test, *p*<0.001). No significant differences were detected between male and female patients (FS autapses: *p* = 0.71; FS-PC synapses: *p* = 0.79). (B,C) No obvious correlation was observed between PT-AR duration and patients' age and time since seizure onset. (D–F) The difference in PT-AR duration occurred regardless of the causes of seizure, occurrence frequency, and seizure duration. Only those patients with seizure occurrence frequency higher than once a week but lower than once a day (1/w≤f<1/d) showed a small AR difference between the two types of connections. Note that patients with longer seizure duration (≥3 min) had larger PT-AR duration than those with shorter seizures (<3 min). * *p*<0.05; ** *p*<0.01; *** *p*<0.001.
(TIF)

Figure S2 Postsynaptic spiking had no effect on AR strength. (A) Example recording from an FS-PC pair. Control: only presynaptic FS neuron was stimulated. Paired firing: Both FS neuron and PC were stimulated simultaneously. (B) Expanded traces (shadowed parts shown in A) for clarity. (C) Group data from 6 FS-PC pairs showing no significant differences in PT-AR duration (left) and total number of events (right) between control and paired firing. In this experiment, APV (50 µM) and CNQX (20 µM) were applied in the bath to block the fast glutamatergic transmission.
(TIF)

Figure S3 Comparing the ratio of PT-AR to total release in FS-PC synapses of control and pilocarpine-treated rats. (A) Calculation of the quanta released during (Train) and after (PT-AR) the train stimulation (40 APs at 200 Hz in FS neurons). The total release is the sum of Train and PT-AR. (B–C) Bar plots of the number of FS-PC pairs versus PT-AR ratio. Note the differences between control and pilocarpine rats. The mean PT-AR ratios for different groups were indicated.
(TIF)

Figure S4 AR evoked by prolonged high-frequency firing. (A) Left, example trace showing prolonged stimulation (300 APs at 150 Hz) in a human FS neuron caused AR at both autaptic and FS-PC synaptic connections. Right, group data indicate that PT-AR frequency at autapses was significantly higher than FS-PC synapses. (B,C) Example FS-PC pair recording in control and pilocarpine-treated rats. (D,E) Group data showing the significant enhancement of PT-AR frequency in model animals (bin size: 200 ms). * *p*<0.05; ** *p*<0.01; *** *p*<0.001.
(TIF)

Figure S5 Changes in AP waveform after the bath application of a low concentration of TTX. (A) Phase plot of the first APs evoked by 500-ms current injections pre and post the application of TTX (100 nM). (B) Changes in AP parameters pre and post TTX (*n* = 9 FS neurons). * *p*<0.05; ** *p*<0.01.
(TIF)

## Abbreviations

AP: action potential
AR: asynchronous release
FS: fast-spiking interneuron
IPSC: inhibitory postsynaptic current
IPSP: inhibitory postsynaptic potential
PC: pyramidal cell
PT-AR: post-train asynchronous release
