# Supplementary figures and images for: Enhancement of Asynchronous Release from Fast-Spiking Interneuron in Human and Rat Epileptic Neocortex
Source: PLoS Biol. 2012 May 8;10(5):e1001324. doi: 10.1371/journal.pbio.1001324 (PMC3348166; doi:10.1371/journal.pbio.1001324)

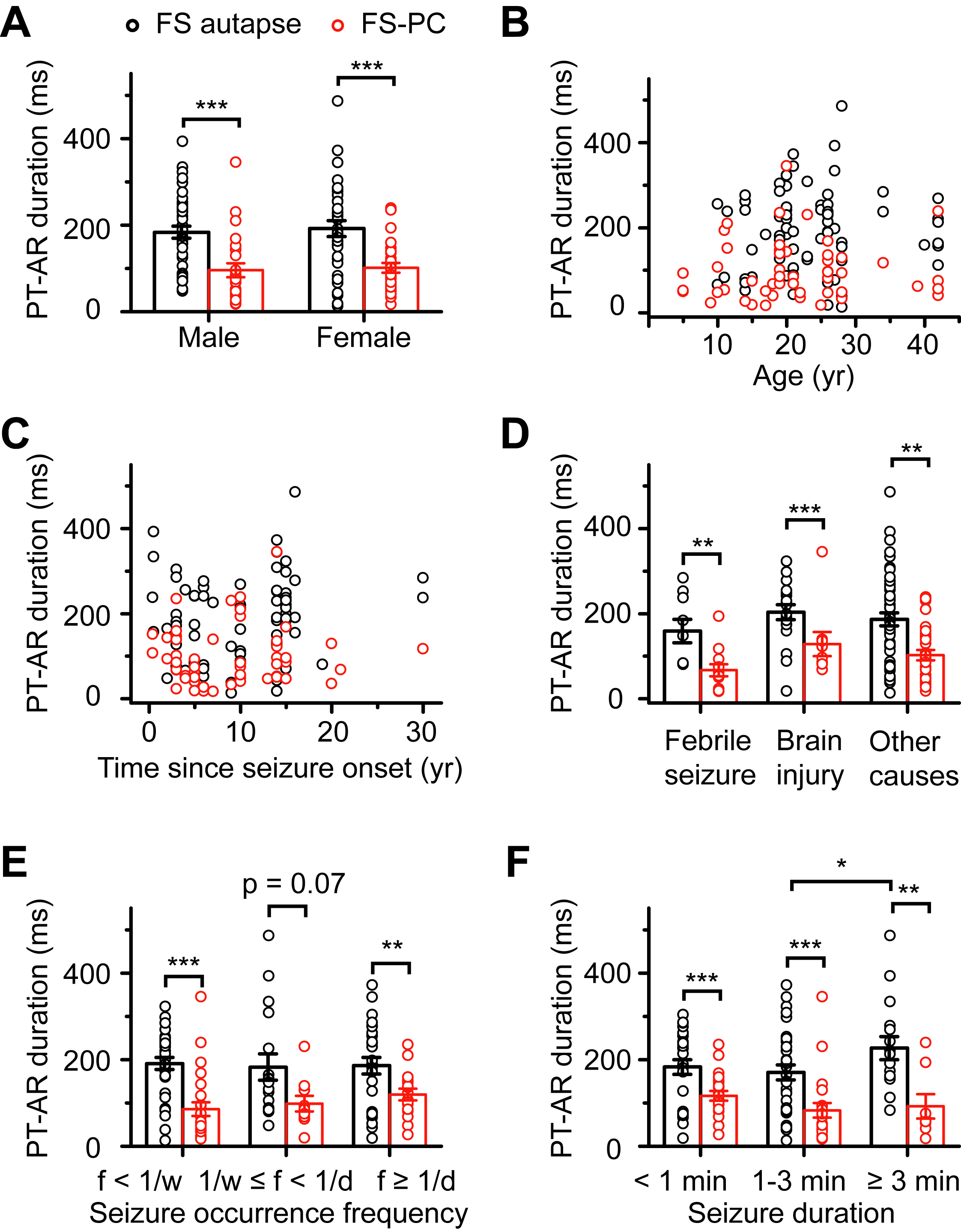

Supplement: Figure S1 — Correlation between PT-AR duration and clinical parameters. (A) Significant difference in AR duration between autaptic and FS-PC connections was observed in both male and female patients (K-S test, p<0.001). No significant differences were detected between male and female patients (FS autapses: p = 0.71; FS-PC synapses: p = 0.79). (B,C) No obvious correlation was observed between PT-AR duration and patients' age and time since seizure onset. (D–F) The difference in PT-AR duration occurred regardless of the causes of seizure, occurrence frequency, and seizure duration. Only those patients with seizure occurrence frequency higher than once a week but lower than once a day (1/w≤f<1/d) showed a small AR difference between the two types of connections. Note that patients with longer seizure duration (≥3 min) had larger PT-AR duration than those with shorter seizures (<3 min). * p<0.05; ** p<0.01; *** p<0.001. (TIF) [file pbio.1001324.s001.tif]

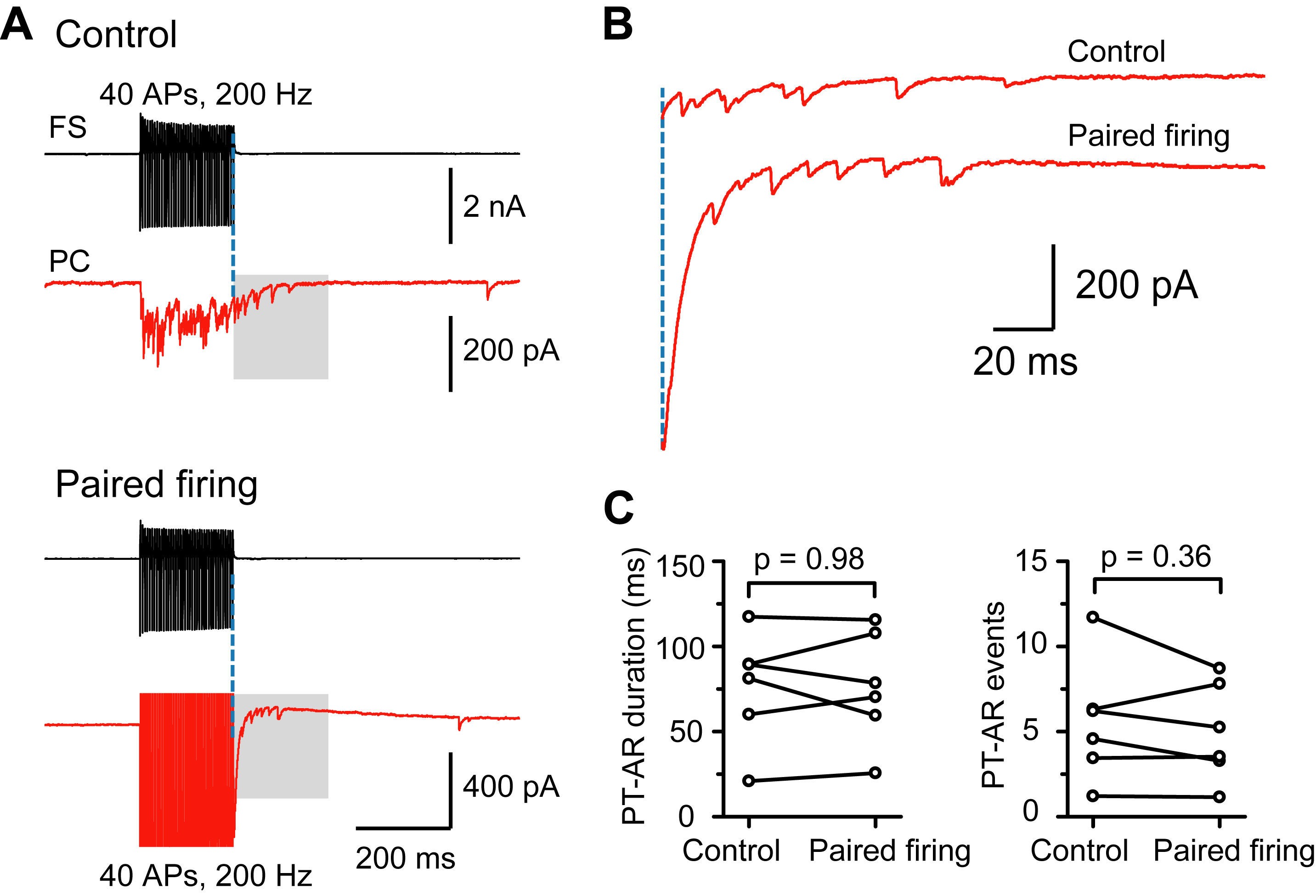

Supplement: Figure S2 — Postsynaptic spiking had no effect on AR strength. (A) Example recording from an FS-PC pair. Control: only presynaptic FS neuron was stimulated. Paired firing: Both FS neuron and PC were stimulated simultaneously. (B) Expanded traces (shadowed parts shown in A) for clarity. (C) Group data from 6 FS-PC pairs showing no significant differences in PT-AR duration (left) and total number of events (right) between control and paired firing. In this experiment, APV (50 µM) and CNQX (20 µM) were applied in the bath to block the fast glutamatergic transmission. (TIF) [file pbio.1001324.s002.tif]

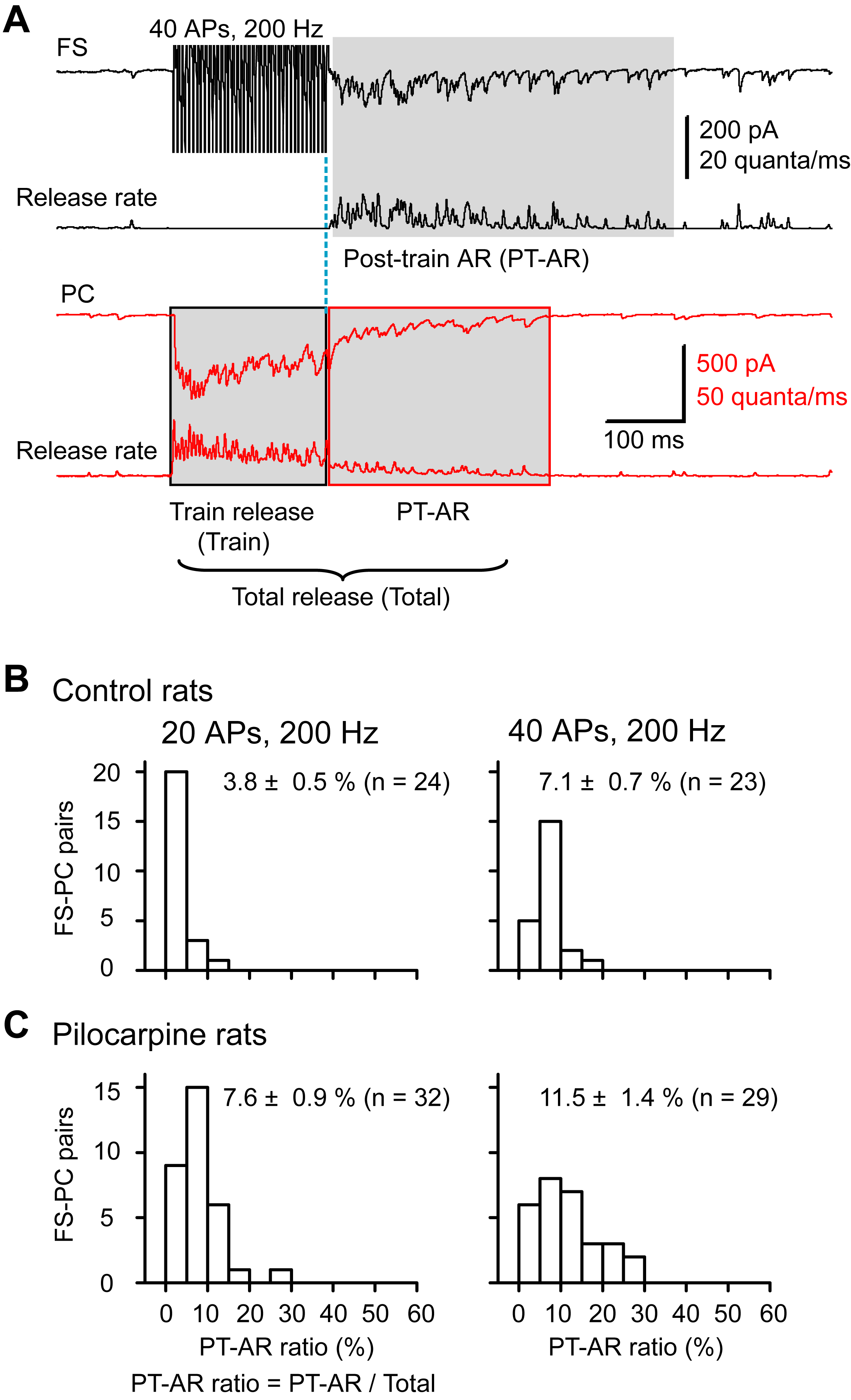

Supplement: Figure S3 — Comparing the ratio of PT-AR to total release in FS-PC synapses of control and pilocarpine-treated rats. (A) Calculation of the quanta released during (Train) and after (PT-AR) the train stimulation (40 APs at 200 Hz in FS neurons). The total release is the sum of Train and PT-AR. (B–C) Bar plots of the number of FS-PC pairs versus PT-AR ratio. Note the differences between control and pilocarpine rats. The mean PT-AR ratios for different groups were indicated. (TIF) [file pbio.1001324.s003.tif]

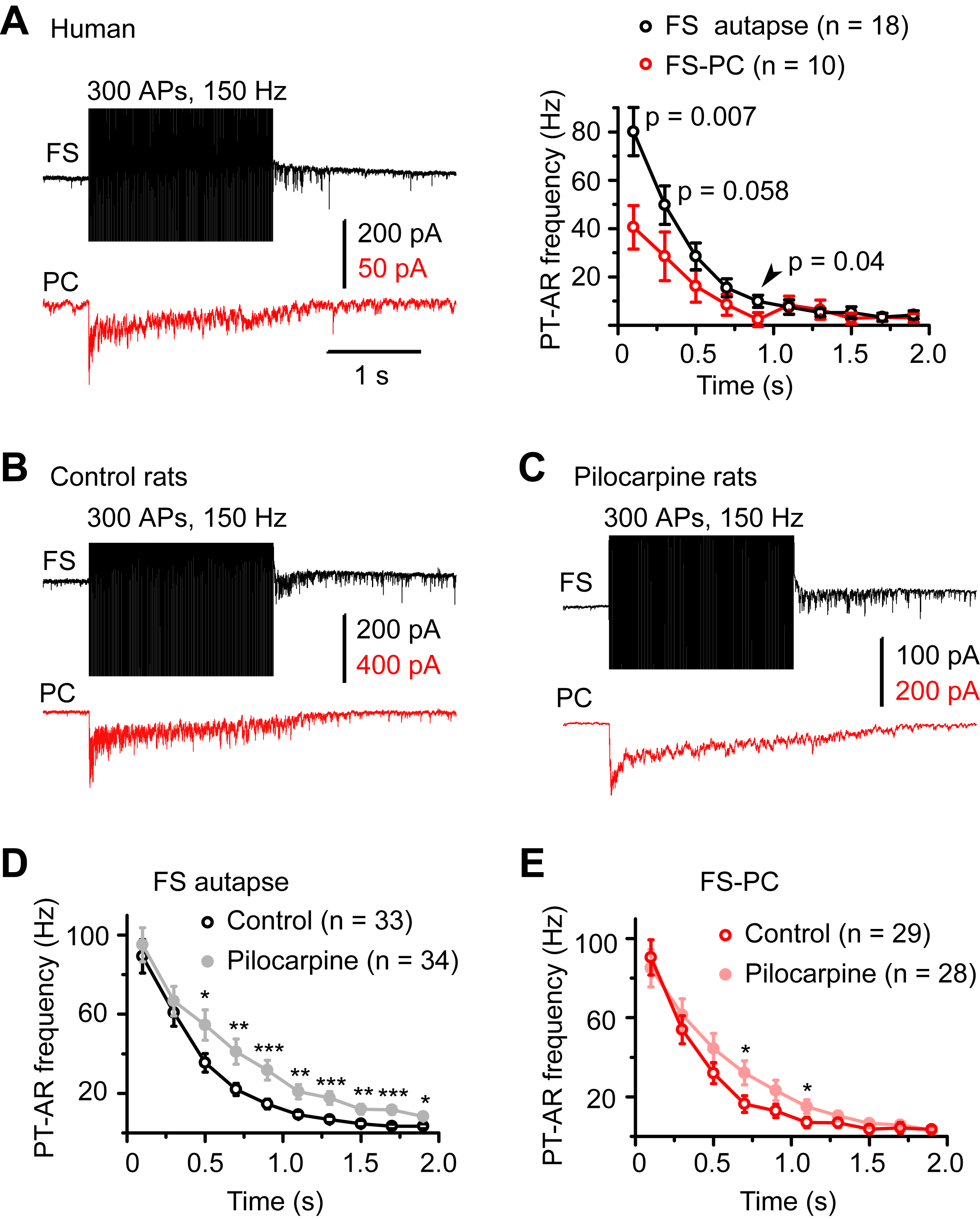

Supplement: Figure S4 — AR evoked by prolonged high-frequency firing. (A) Left, example trace showing prolonged stimulation (300 APs at 150 Hz) in a human FS neuron caused AR at both autaptic and FS-PC synaptic connections. Right, group data indicate that PT-AR frequency at autapses was significantly higher than FS-PC synapses. (B,C) Example FS-PC pair recording in control and pilocarpine-treated rats. (D,E) Group data showing the significant enhancement of PT-AR frequency in model animals (bin size: 200 ms). * p<0.05; ** p<0.01; *** p<0.001. (TIF) [file pbio.1001324.s004.tif]

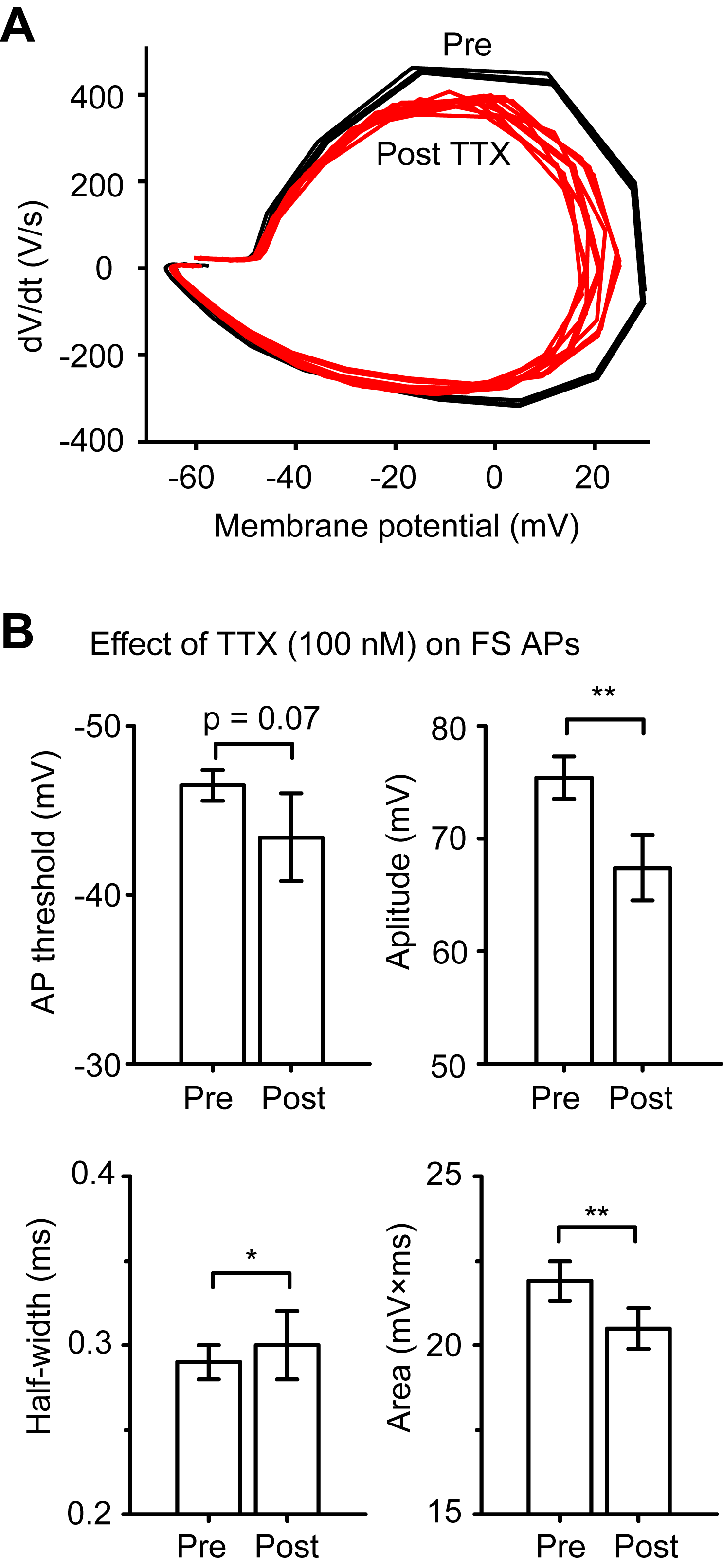

Supplement: Figure S5 — Changes in AP waveform after the bath application of a low concentration of TTX. (A) Phase plot of the first APs evoked by 500-ms current injections pre and post the application of TTX (100 nM). (B) Changes in AP parameters pre and post TTX (n = 9 FS neurons). * p<0.05; ** p<0.01. (TIF) [file pbio.1001324.s005.tif]
